# Using Middle Cerebral Artery Doppler Ultrasound to Predict Clinical Chorioamnionitis After Preterm Prelabor Rupture of Membranes

**DOI:** 10.7759/cureus.41508

**Published:** 2023-07-07

**Authors:** Julie M Leizer, Ammoura M Ibrahim, Llewellyn A Foulke, Kate A Tauber, Paul Feustel, Craig M Zelig

**Affiliations:** 1 Obstetrics and Gynecology, Robert Wood Johnson (RWJ) Barnabas Health, Eatontown, USA; 2 Pathology, University of Miami Miller School of Medicine, Miami, USA; 3 Pathology, Albany Medical Center, Albany, USA; 4 Neonatology, Albany Medical Center, Albany, USA; 5 Neuroscience and Experimental Therapeutics, Albany Medical College, Albany, USA; 6 Obstetrics and Gynecology, Albany Medical Center, Albany, USA

**Keywords:** rupture of membranes, pprom, mca doppler, ultrasound, chorioamnionitis

## Abstract

Background: In neonates, blood flow to the brain as measured by peak systolic velocity (PSV) in the middle cerebral artery (MCA) is altered in pregnancies affected by chorioamnionitis.

Objective: We aim to determine whether PSV and other measures of flow in the MCA in the fetus are altered prior to the development of clinical chorioamnionitis following preterm prelabor rupture of membranes (PPROM).

Methods: This was a prospective observational study. Fifty patients from one institution were recruited after being diagnosed with PPROM between 23 weeks zero days and 33 weeks six days gestation. We performed measurements of the PSV in the fetal MCA on a weekly basis following PPROM and used the value taken closest to the time of delivery for our statistical analysis. The primary outcome assessed was clinical chorioamnionitis, and the exposure of interest was MCA PSV. Additional independent variables of interest were other Doppler measures of the MCA. Secondary outcomes included histological chorioamnionitis and other measures of neonatal health, including sepsis, days in the neonatal intensive care unit (NICU), and death.

Results: Of the 50 patients recruited to our study, eight (16%) developed clinical chorioamnionitis, similar to previously reported values in the general population. The PSV in the MCA was not significantly associated with the development of clinical chorioamnionitis. However, an elevated MCA pulsatility index (PI), a measure of resistance to flow, was associated with a higher probability of developing clinical chorioamnionitis.

Conclusion: There does not appear to be a difference in the PSV of the MCA of fetuses in pregnancies following PPROM with impending chorioamnionitis. However, elevated PI in the MCA could be a marker of impending chorioamnionitis in PPROM. Larger studies are needed to confirm these findings.

## Introduction

Chorioamnionitis is an infection of the intra-amniotic cavity that complicates a small fraction of pregnancies. While chorioamnionitis usually occurs during labor, it can also occur in the antepartum period following preterm prelabor rupture of membranes (PPROM) [1]. The clinical management of pregnancies complicated by PPROM prior to 34 weeks gestation includes the short-term use of tocolytics as needed to allow the administration of corticosteroids, antibiotics to delay the onset of labor to reduce the increased neonatal morbidity associated with prematurity, and magnesium sulfate to reduce the risk for cerebral palsy if delivery is imminent prior to 32 weeks gestation [2-5]. Chorioamnionitis has been associated with adverse maternal and neonatal outcomes including higher rates of maternal wound infections, endometritis, and sepsis, as well as increased rates of intraventricular hemorrhage (IVH), cerebral palsy, and neurodevelopmental impairment in the neonate [6-8]. For that reason, induction of labor is undertaken at the first signs of chorioamnionitis following PPROM, regardless of the gestational age. Diagnosis of chorioamnionitis is based on clinical signs such as maternal fever, fundal tenderness, and tachycardia. There is currently no laboratory or radiological findings that can predict chorioamnionitis with sufficient sensitivity and specificity to guide delivery decisions. Doppler ultrasound (US) of various fetal vessels is used clinically to diagnose and manage pregnancy complications such as fetal anemia and fetal growth restriction [9-11]. By contrast, the ability of Doppler US to diagnose or predict the development of chorioamnionitis has not been demonstrated nor applied to clinical practice. In a retrospective study of pregnancies complicated by PPROM, no association was seen between the pulsatility index (PI) in the middle cerebral artery (MCA) and suspected chorioamnionitis in a subset of patients with PPROM and fetal growth restriction [12]. In a prospective study by Carroll et al. [13], no association was found between Doppler measurements of the fetal umbilical or middle cerebral circulation and intra-amniotic infection diagnosed with amniocentesis or fetal bacteremia diagnosed with cordocentesis.

However, in a novel study of neonates born following PPROM, peak systolic flows in the MCA as measured with Doppler US were found to be altered in those cases diagnosed with histological chorioamnionitis [14]. We hypothesized that a similar phenomenon could be happening in human pregnancies. Specifically, in pregnancies complicated by PPROM and intrauterine infection, the maximum velocity of flow to the fetal brain could be increased, resulting in higher peak flow in the MCA as measured with Doppler ultrasound. In addition to peak systolic velocity (PSV), we also explored the association of various additional measures of MCA flow in the fetal circulation including systolic-to-diastolic ratio (S/D) and pulsatility index (PI) with the development of both clinical and histological chorioamnionitis.

## Materials and methods

This was a prospective, observational study of singleton pregnancies complicated by PPROM. This study was approved by the Institutional Review Board (IRB) at Albany Medical Center (IRB study number: 5008). Study participants were recruited from patients admitted to our hospital with a diagnosis of preterm prelabor rupture of membranes (PPROM) for whom expectant management was planned. Pregnancies affected by conditions that precluded expectant management or that could affect MCA Dopplers were excluded from the study. Inclusion criteria were maternal age of at least 18 years of age, PPROM between 23 weeks zero days and 33 weeks six days gestation, and singleton gestation. Exclusion criteria included active labor or chorioamnionitis at the time of admission, known major fetal anomalies, maternal alloimmunization, known fetal growth restriction, and multiple gestation pregnancy. Eligible women were identified on admission to the Labor and Delivery unit and consented to participate in the study.

**Figure 1:**
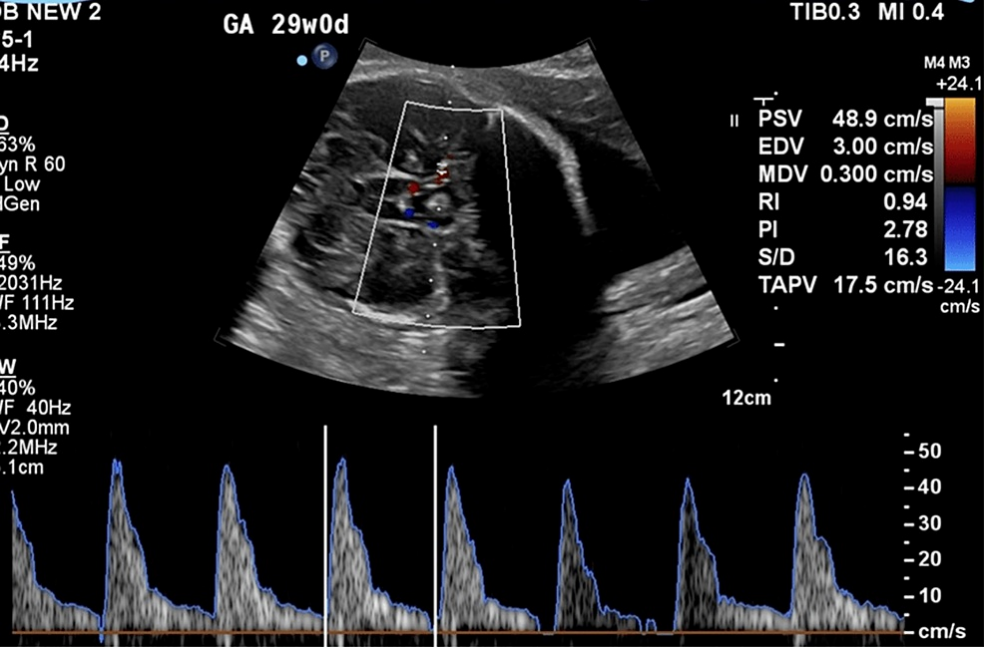
MCA Doppler Measurements MCA - middle cerebral artery, PSV - peak systolic velocity, PI - pulsatility index

While all subjects received the same interventions, they were separated into study groups based on whether or not they developed clinical chorioamnionitis during their hospitalization for PPROM. A secondary analysis separated patients into study groups based on the identification of histological chorioamnionitis on pathologic examination of their placentas following delivery. All study participants received standard treatment for PPROM including prolonged fetal monitoring on admission, daily nonreactive stress tests, latency antibiotics, weekly ultrasound assessments including biophysical profile (BPP), antenatal steroids, and magnesium sulfate for neuroprotection if the estimated gestational age (EGA) was less than 32 weeks zero days and delivery was believed to be imminent. In addition to the standard care received by all PPROM patients, study participants received additional measurements during their weekly US to include the measurement of PSV in the fetal MCA. These MCA Doppler measurements were obtained using a standardized technique by sonographers trained and experienced in its use (Figure 1). This US protocol demonstrated excellent inter- and intra-observer variability in a prospective study by Mari et al. [15]. In addition, patients in the study received Doppler measurements of the pulsatility index (PI = (PSV - EDV) / TAV, where PSV is the peak systolic velocity, EDV is the end-diastolic velocity, and TAV is the time-averaged velocity) and systolic-to-diastolic (S/D) ratio in the MCA. MCA Doppler measurements are routinely obtained in our practice for indications other than PPROM and all of our sonographers are very experienced in their performance. Indications for delivery included clinical evidence of chorioamnionitis, non-reassuring fetal testing, active labor, severe preeclampsia, placental abruption, umbilical cord prolapse, fetal demise, or attainment of the gestational age of 34 weeks zero days. The diagnosis of non-reassuring fetal testing was left to the discretion of the attending physician based on their interpretation of fetal external monitoring and biophysical profiles. In general, a fetal tracing with recurrent decelerations unresponsive to conservative measures or a nonreactive non-stress test (NST) in combination with a BPP of less than 6 out of 10 would prompt consideration for delivery. Doppler measurements in the MCA were not used to make delivery decisions. A data sheet was filled out for each study participant including all ultrasound measurements, maternal and neonatal clinical outcomes, and results of histological examination of the placenta.

Statistical analysis

A sample size of 50 patients was calculated using a two-sided alpha level of 0.05 and 80% power to detect a 30% difference in the PSV of MCA Doppler between patients with chorioamnionitis and those without chorioamnionitis based on a previous study performed in neonates born following chorioamnionitis [14]. A rate of clinical chorioamnionitis of 15% was assumed based on historical averages [1-5].

The primary outcome was clinical chorioamnionitis, and the exposure of interest was PSV in the MCA. Additional exposures that were evaluated included PI and S/D ratio in the MCA. Secondary outcomes of interest included histological chorioamnionitis and various neonatal outcomes. Clinical chorioamnionitis was defined as the presence of uterine tenderness and/or purulent discharge with any two of the following: temperature of 100.4°F or more, maternal tachycardia (>120 bpm), white blood cell (WBC) count > 18,000 cells/mm^3^, or fetal tachycardia (>160 bpm) [1,2,7]. Placentas were evaluated by a trained pathologist (LF) for evidence of histological chorioamnionitis based on the infiltration of the chorioamnion plate by polymorphonuclear leukocytes on high-power field microscopy using standardized criteria [16]. Clinical neonatal outcomes were extracted from the electronic inpatient chart for each patient in the study and recorded on a data sheet by a neonatologist (KT).

Univariate odds ratios (ORs) were calculated for the development of clinical chorioamnionitis as a function of PSV in the MCA, our primary outcome. Univariate ORs were also calculated for the development of clinical chorioamnionitis as a function of our secondary Doppler ultrasound parameters: S/D and PI in the MCA. PSV in the MCA was also assessed as multiples of the median (MOMs) to account for variability in the MCA PSV at different gestational ages. The same analysis was carried out for the development of histological chorioamnionitis. These results were expressed as ORs with 95% confidence intervals (CIs). Next, binary logistic regression was carried out utilizing forced entry of the individual ultrasound variables. Variance inflation factors (VIFs) were calculated to assess for multicollinearity. Measures of neonatal health were compared between patients developing chorioamnionitis and those who did not. For continuous variables, odds ratios are per unit change in the independent variable. Minitab Statistical Software was used. Continuous variables for neonatal outcomes are expressed as mean and standard deviation (SD) with t-test used to test for statistical significance.

## Results

Of the 50 patients recruited to our study, eight (16%) developed clinical chorioamnionitis, similar to previously reported values in the general population [5,17]. The MCA PSV was not significantly different in patients who developed chorioamnionitis compared to those who did not. Other Doppler measures in the MCA were also not statistically different between these two groups. However, the MCA pulsatility index (PI) was significantly higher in patients who developed clinical chorioamnionitis. There was significant collinearity observed, specifically between MCA PI and MCA S/D with VIF values greater than 10. As a result, only the univariate analysis is presented. These results are shown in Table 1. Among the patients who developed clinical chorioamnionitis, gestational age at diagnosis and delivery was earlier and birth weight was less, but the latency period was not significantly different. Neonatal outcomes that were significantly worse in patients who developed clinical chorioamnionitis included sepsis and the need for intubation. Other neonatal complications such as IVH, bronchopulmonary dysplasia (BPD), and neonatal demise (NND) demonstrated a non-statistically significant trend toward higher incidence in pregnancies affected by clinical chorioamnionitis. When these same factors were compared between patients who developed histological chorioamnionitis versus those who did not, only birth weight was significantly different. These results are shown in Table 2.

**Table 1:** Association Between Middle Cerebral Artery Dopplers and Clinical Chorioamnionitis ^a^Two-tailed t-test ^b^Fisher’s exact test (no CI for AOR because no subjects with chorioamnionitis had MCA ≥ 1.29 MOMs) ^c^Univariate odds ratio AOR - adjusted odds ratio, CI - confidence interval, MCA - middle cerebral artery, MOMs - multiples of the median, OR - odds ratio, PI - pulsatility index, PSV - peak systolic velocity, SD - standard deviation, S/D - systolic-to-diastolic ratio

Measure (Mean (SD))	Clinical Chorioamnionitis Absent	Clinical Chorioamnionitis Present	p	OR (95% CI)^c^	p
PSV of MCA^a^	48.5 (10.2)	41.8 (8.6)	0.08	0.93 (0.85, 1.01)	0.095
S/D MCA^a^	4.97 (1.45)	7.45 (4.13)	0.33	1.52 (0.99, 2.34)	0.054
PI MCA^a^	1.88 (0.51)	2.73 (1.30)	0.14	3.41 (1.15, 10.09)	0.027
MOM MCA^a^	1.13 (0.22)	1.11 (0.11)	0.71	0.63 (0.016, 25.56)	0.81
MOM MCA ≥ 1.29^b^	26.8%	0%	0.17	0	0.96

**Table 2:** Clinical Outcomes Associated With Clinical and Histological Chorioamnionitis BPD - bronchopulmonary dysplasia, EGA US - estimated gestational age at the time of ultrasound, EGA delivery - estimated gestational age at the time of delivery, IVH - intraventricular hemorrhage, NEC - necrotizing enterocolitis, NICU - neonatal intensive care unit, NND - neonatal demise, PPHN - persistent pulmonary hypertension of the newborn, SD - standard deviation

Value (Mean (SD))	Clinical Chorioamnionitis Absent	Clinical Chorioamnionitis Present	p	Histological Chorioamnionitis Absent	Histological Chorioamnionitis Absent	p
	N=42	N=8		N=14	N=36	
EGA US	30.79 (2.29)	27.63 (2.83)	0.001	31.13 (2.23)	29.94 (2.73)	0.17
EGA delivery	31.48 (2.36)	27.88 (2.64)	<0.001>	31.93 (2.37)	30.51 (2.77)	0.09
Latency (days)	12.70 (13.01)	20.00 (22.55)	0.39	16.87 (18.30)	12.59 (13.25)	0.35
Apgar (1 minute)	6.14 (2.70)	4.13 (2.53)	0.06	6.13 (3.02)	5.70 (2.67)	0.62
Apgar (5 minutes)	7.71 (2.12)	6.38 (2.45)	0.12	7.60 (2.35)	7.46 (2.17)	0.84
Birth weight (g)	1,803 (411.8)	1,255 (432)	0.001	1,962 (408)	1,124.5 (445.2)	<0.001>
Days in NICU	34.41 (20.55)	46.4 (35.3)	0.38	32 (22.92)	38.11 (23.86)	0.42
Sepsis	3 (7.1)	4 (50)	0.009	0 (0)	7 (19.4)	0.08
IVH	3 (7.1)	3 (37.5)	0.07	1 (7.1)	5 (13.9)	0.54
NEC	1 (2.4)	0 (0)	0.07	0 (0)	1 (2.8)	>0.99
Intubation	5 (11.9)	4 (50)	0.03	1 (7.1)	7 (19.4)	0.54
PPHN	1 (2.4)	1 (12.5)	0.59	0 (0)	2 (5.6)	>0.99
BPD	5 (11.9)	3 (37.5)	0.09	2 (14.3)	6 (16.7)	>0.99
NND	0 (0)	2 (25%)	0.08	0 (0)	2 (5.6)	0.26

## Discussion

Inflammatory processes such as chorioamnionitis are strongly associated with brain injury in premature neonates. One of the primary mechanisms for this phenomenon is the loss of autoregulation of cerebral blood flow to the immature fetal brain. The resultant fluctuations in cerebral perfusion caused by this loss of autoregulation in conjunction with infiltration of inflammatory cells are thought to compromise the fetal blood-brain barrier. The resultant intraventricular hemorrhage can cause periventricular leukomalacia and diffuse white matter injury, both of which can be observed in premature neonates following PPROM and chorioamnionitis [6-8,17].

Chorioamnionitis is an infection of the placenta, and the resultant inflammation would be expected to impair oxygen delivery by the placenta to the fetus. In response to placental insufficiency, fetuses redistribute their blood flow to vital organs including the brain by reducing the resistance in the arteries supplying these vital organs. Evidence for this phenomenon was observed in growth-restricted fetuses in pregnancies affected by placental insufficiency. In those pregnancies, there was decreased resistance to cerebral blood flow, which resulted in higher peak systolic velocities (PSV) of the MCA Dopplers [9].

Our study failed to demonstrate an association between MCA PSV and clinical chorioamnionitis, which would have mirrored the results of the study by Koch et al. [14] in neonates. However, we did observe an association between increased MCA PI and the development of clinical chorioamnionitis. This is opposite from what would be expected based on the phenomenon of “brain sparing” seen in hypoxic fetuses, that is, the preferential shunting of fetal blood to the vital organs (brain, heart, and adrenal glands) and away from visceral organs (liver and kidney). Specifically, we would have expected a decrease in MCA PI, reflecting a drop in resistance to brain blood flow (brain sparing) in response to fetal compromise caused by chorioamnionitis. If the increase in MCA PI we observed is a true phenomenon, it would imply a different mechanism regulating blood flow to the fetal brain during infectious processes. Perhaps the increased resistance to flow is a protective mechanism for the fetal brain. Theoretically, this makes sense as one of the principal mechanisms postulated to cause cerebral palsy in premature infected neonates is loss of cerebral blood flow autoregulation with rapid volume expansion causing damage to the delicate germinal matrix in the immature fetal brain [17]. The trend toward lower MCA PSV in pregnancies with chorioamnionitis observed in our study supports this theory.

In our study, MCA PSV was our primary variable of interest due to the findings in a previous neonatal study that demonstrated changes in MCA PSV of neonatal Dopplers associated with chorioamnionitis [4]. In addition, MCA PSV is a standard measurement obtained routinely during ultrasound to assess for fetal anemia at high-risk obstetrics (OB) referral centers such as our own. While previous studies failed to demonstrate an association between MCA PI and chorioamnionitis, those studies included PPROM patients with the concomitant diagnosis of fetal growth restriction, a condition known to be associated with lower MCA PI. Therefore, the finding of higher MCA PI measurements in our patients with chorioamnionitis is not inconsistent with the previous MCA PI study because our study excluded patients with known fetal growth restriction [13]. A second study that also failed to demonstrate an association between MCA PI and chorioamnionitis used laboratory criteria for diagnosing infection, not clinical criteria as was used in our study and that is used in actual practice [12].

MCA PSV and, to a lesser extent, MCA PI are gestational age-dependent. MCA PSV increases with more advanced gestational ages, while MCA PI decreases with more advanced gestational ages, reflecting decreased resistance to flow in the growing placenta. To ensure that gestational age did not skew our results, we also calculated MCA PSV as multiples of the median for gestational age. While similar gestational age-based averages were not used when comparing MCA PI values, the reference curve for MCA PI is very flat over the gestational age range of our patients. The difference in reference values for mean MCA PI over the gestational age range of our study is fivefold less than the actual difference we observed between the two groups in our study. Nonetheless, in future studies that study MCA PI as the primary variable of interest, measurements normalized to gestational age-based means should be used for better precision.

The current standard of care for pregnancies affected by PPROM is expectant management until 34 weeks zero days to 36 weeks six days. The goal of this strategy is to reduce neonatal morbidity and mortality associated with delivery at earlier gestational ages. One of the risks of expectant management is the development of chorioamnionitis, primarily through the vertical ascent of the normal flora of the vagina no longer isolated from the intrauterine cavity by the fetal membranes. Given the well-known association of chorioamnionitis with adverse neonatal outcomes such as cerebral palsy, the ability to identify markers of preclinical disease could be used to time delivery following PPROM prior to the development of overt chorioamnionitis, potentially improving neonatal and maternal outcomes.

Limitations of this study include its sample size based on a single predictor variable (MCA PSV) and a single outcome (clinical chorioamnionitis). As a result, the number of patients with clinical chorioamnionitis precluded testing multiple independent variables simultaneously. The multicollinearity we observed in our data set precluded the development of a predictive model based on multiple ultrasound parameters. Also, the study is from a single center and may not be broadly generalizable.

## Conclusions

Doppler measurement of the PSV in the fetal MCA blood vessels did not predict chorioamnionitis in pregnant patients following PPROM. However, an elevated fetal MCA PI value, a measure of vascular resistance, was associated with increased development of clinical chorioamnionitis in our study.

Doppler interrogation of the MCA blood vessels in the fetus of a pregnant patient following PPROM may someday prove to be a useful tool for predicting impending chorioamnionitis. This would allow more timely delivery in these pregnancies, i.e., at later gestational ages prior to the development of chorioamnionitis, reducing the complications of infection in the mother and neonate and morbidities of prematurity in the neonate.

## References

[1] Mercer BM (2003). Preterm premature rupture of the membranes. Obstet Gyncol.

[2] Higgins RD, Saade G, Polin RA (2016). Evaluation and management of women and newborns with a maternal diagnosis of chorioamnionitis: summary of a workshop. Obstet Gynecol.

[3] Yudin MH, van Schalkwyk J, Van Eyk N (2017). No. 233-Antibiotic therapy in preterm premature rupture of the membranes. J Obstet Gynaecol Can.

[4] Kenyon SL, Taylor DJ, Tarnow-Mordi W, ORACLE Collaborative Group (2001). Broad-spectrum antibiotics for preterm, prelabour rupture of fetal membranes: the ORACLE I randomised trial. ORACLE Collaborative Group. Lancet.

[5] Garite TJ, Freeman RK (1982). Chorioamnionitis in the preterm gestation. Obstet Gynecol.

[6] Dexter SC, Pinar H, Malee MP, Hogan J, Carpenter MW, Vohr BR (2000). Outcome of very low birth weight infants with histopathologic chorioamnionitis. Obstet Gynecol.

[7] Ramsey PS, Lieman JM, Brumfield CG, Carlo W (2005). Chorioamnionitis increases neonatal morbidity in pregnancies complicated by preterm premature rupture of membranes. Am J Obstet Gynecol.

[8] Horvath B, Grasselly M, Bodecs T, Boncz I, Bodis J (2012). Histological chorioamnionitis is associated with cerebral palsy in preterm neonates. Eur J Obstet Gynecol Reprod Biol.

[9] Mari G, Hanif F, Kruger M, Cosmi E, Santolaya-Forgas J, Treadwell MC (2007). Middle cerebral artery peak systolic velocity: a new Doppler parameter in the assessment of growth-restricted fetuses. Ultrasound Obstet Gynecol.

[10] Mari G, Deter RL, Carpenter RL (2000). Noninvasive diagnosis by Doppler ultrasonography of fetal anemia due to maternal red-cell alloimmunization. Collaborative Group for Doppler Assessment of the Blood Velocity in Anemic Fetuses. N Engl J Med.

[11] (2018). ACOG Practice Bulletin No. 192: Management of Alloimmunization During Pregnancy. Obstet Gynecol.

[12] Aviram A, Quaglietta P, Warshafsky C (2020). Utility of ultrasound assessment in management of pregnancies with preterm prelabor rupture of membranes. Ultrasound Obstet Gynecol.

[13] Carroll SG, Papaioannou S, Nicolaides KH (1995). Doppler studies of the placental and fetal circulation in pregnancies with preterm prelabor amniorrhexis. Ultrasound Obstet Gynecol.

[14] Koch FR, Wagner CL, Jenkins DD (2014). Sex differences in cerebral blood flow following chorioamnionitis in healthy term infants. J Perinatol.

[15] Mari G, Abuhamad AZ, Cosmi E, Segata M, Altaye M, Akiyama M (2005). Middle cerebral artery peak systolic velocity: technique and variability. J Ultrasound Med.

[16] Roberts DJ, Baergen RN, Boyd TK (2023). Criteria for placental examination for obstetrical and neonatal providers. Am J Obstet Gynecol.

[17] Romantsik O, Bruschettini M, Ley D (2019). Intraventricular hemorrhage and white matter injury in preclinical and clinical studies. Neoreviews.

